# Parkinson’s disease medication state and severity assessment based on coordination during walking

**DOI:** 10.1371/journal.pone.0244842

**Published:** 2021-02-17

**Authors:** Carla Agurto, Stephen Heisig, Avner Abrami, Bryan K. Ho, Vittorio Caggiano

**Affiliations:** 1 IBM Research - Healthcare and Life Sciences, Yorktown Heights, Yorktown, New York, United States of America; 2 Department of Neurology, Boston, Massachusetts, United States of America; Istituto Italiano di Tecnologia, ITALY

## Abstract

Walking is a complex motor function requiring coordination of all body parts. Parkinson’s disease (PD) motor signs such as rigidity, bradykinesia, and impaired balance affect movements including walking. Here, we propose a computational method to objectively assess the effects of Parkinson’s disease pathology on coordination between trunk, shoulder and limbs during the gait cycle to assess medication state and disease severity. Movements during a scripted walking task were extracted from wearable devices placed at six different body locations in participants with PD and healthy participants. Three-axis accelerometer data from each device was synchronized at the beginning of either left or right steps. Canonical templates of movements were then extracted from each body location. Movements projected on those templates created a reduced dimensionality space, where complex movements are represented as discrete values. These projections enabled us to relate the body coordination in people with PD to disease severity. Our results show that the velocity profile of the right wrist and right foot during right steps correlated with the participant’s total score on the gold standard Unified Parkinson’s Disease Rating Scale (UPRDS) with an r^2^ up to 0.46. Left-right symmetry of feet, trunk and wrists also correlated with the total UPDRS score with an r^2^ up to 0.3. In addition, we demonstrate that binary dopamine replacement therapy medication states (self-reported ‘ON’ or ‘OFF’) can be discriminated in PD participants. In conclusion, we showed that during walking, the movement of body parts individually and in coordination with one another changes in predictable ways that vary with disease severity and medication state.

## Introduction

The generation of commands to initiate and coordinate whole body movements during walking [1,2] is based on an interaction of cortical and subcortical structures (including basal ganglia and brainstem) [3]. Dopaminergic deficiency in the basal ganglia associated with Parkinson’s disease (PD) generates canonical motor signs such as rigidity, bradykinesia, dystonia, and impaired balance which affect movements including walking [4]. The ability to control movements in PD [5] is evaluated in a clinical setting—once or twice per year—as part of an extensive semi-quantitative motor and cognitive assessment of disease progression performed by a movement disorder specialist according to the Movement Disorder Society Unified Parkinson’s Disease Rating Scale (MDS-UPDRS, 2008 version) [6]. Motor impairment is evaluated in part 3 of the UPDRS. Gait and other tasks are scored (with a value between 0–4) by the specialist based on the observation of the task. Thus, the results vary with the training of the physician [7] and are not objectively captured. Tools to assist physicians in quantification and analysis of movement are currently limited to expensive gait labs but they are not part of a standardized process. The possibility of using inexpensive technology and computational methods to measure and quantify whole body motor behavior will allow objective quantification of disease state phenomenology in normal clinical settings.

Although coordination between limbs is not explicitly assessed in the standardized MDS-UPDRS test, it is well known that both upper and lower limb coordination during walking are affected in people with PD both in natural [8,9] and treadmill walking [10]. Typically, due to various PD related impairments, posture and arm swing become asymmetric [8,11–13]. Different laboratories have assessed and quantified these impairments using high definition motion capture analysis and electromyographic recordings [9,14]. These methods are restricted to complex and expensive research laboratories settings, which makes them unsuitable to monitor participants outside the clinic and to understand the effects of medication and interventions on the progression of the disease [4,5] in a more typical setting [15].

Outside the clinical setting, wearable technology [16] offers a solution for continuous monitoring of people with PD [15,17,18]. Gait has been characterized and measured from the trunk [19,20], and feet [21] (see Del Din et al. for a comprehensive review [20]). Various techniques have quantified features of body movement based on one [19,21–25] or multiple sensors attached to different body locations [19,26,27]. Typically, studies have used descriptive features of the signal i.e. discrete measurements such as frequency, stride length, entropy, arm swing, etc. to characterize PD. Those discrete measurements provide a snapshot of step or body coordination, which although easy to understand, do not capture most of the temporal information of how the whole body moves during the walking task e.g. left-right symmetry, limb/core coordination etc. Also, only a few of those discrete analyses looked into measurements of left versus right symmetry [19,27], and step coordination [28]. Recently a study measured coordination between body parts as the variability of the maximum angular extention [29] but no study has looked into overall body coordination during individual steps. We believe that a measurement built from temporal profiles of whole-body readjustment at each step is not only closer to the way a specialist intuitively evaluates patient movements, but it can also enable rating with quantitative granularity.

In this work, we propose a novel method which characterizes the temporal profile of movements across body parts rather than single point metrics to characterize PD. We used data acquired from different parts of the body starting at the point of weight redistribution i.e. during whole body adjustment following a step. By analyzing this temporal profile, we aim to answer the following questions: (i) are the individual movements in different parts of the body during walking affected by disease severity or drug intake in people with PD? (ii) is the symmetry of the body parts during walking affected by either of these factors?

## Results

### Single body part kinematics relative to PD severity

**Fig 1:**
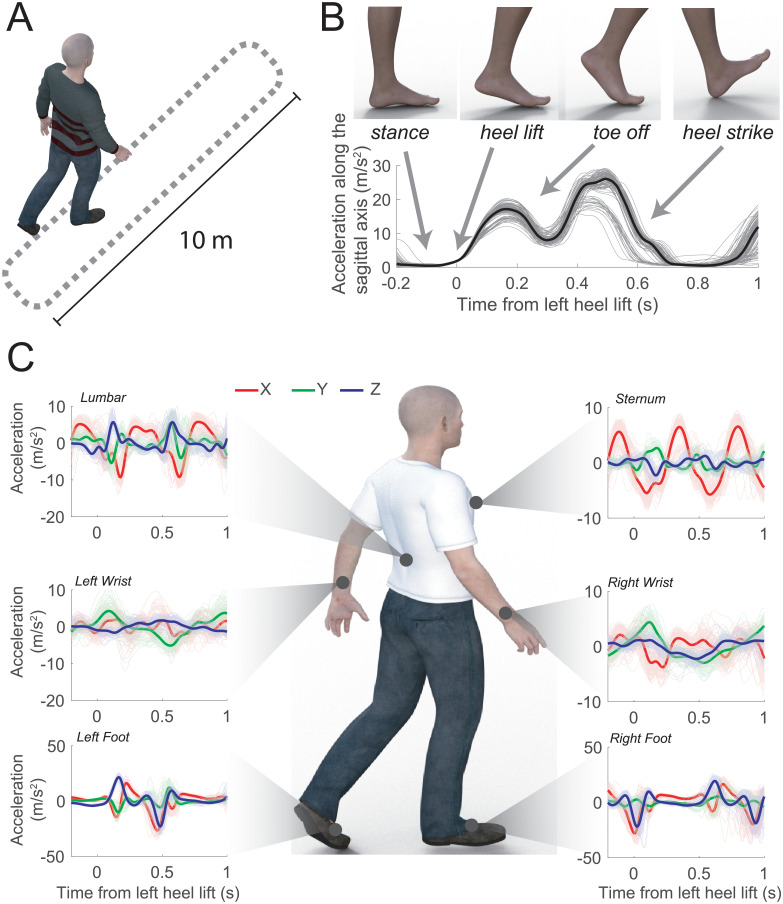
Walking task and movement trajectories aligned at step start. **A**–Walking task route. **B**–Absolute value of acceleration traces along frontal and vertical axis during steps, aligned with heel lift off. **C—**Accelerations recorded at different body parts aligned to left heel lift off events (see S1 Fig for location of sensors and orientations).

To test how body part coordination during walking related to disease severity we collected acceleration information at the feet, wrist, lumbar and sternum during unconstrained walking (see Methods). Those positions provided overall information for movements at lower and upper extremities, core (lumbar) and trunk (sternum, see Figs 1 and S1). We used step events as the start point for the whole-body coordination (see Methods and Fig 1B and 1C) and built a reduced dimensionality space (RDS) at each step (see Methods) to summarize acceleration, velocity and time to a three-dimensional value (see S2 Fig).

**Fig 2:**
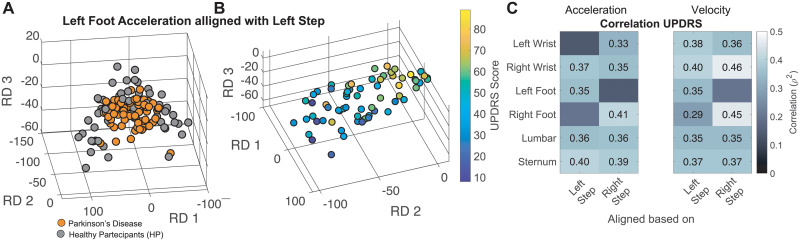
Movements in participants with PD diverge from healthy movements proportional to disease severity. **A**–Projection of healthy movement templates in healthy volunteers HV (gray) and PD (orange) participants. **B**–Projection of age matched healthy movement templates in gray and PD participants color coded according to the UPDRS score. **C**- Results of a linear regression model applied to the 3D of the RDS projections against the UPDRS score for each sensor aligned either with left or right step. The same analysis was repeated both for acceleration (left) and velocity (right). Only values with a p < 0.05 after correction for multiple comparisons (24: 6 sensors x 2 triggers x 2 conditions—velocity and speed) with Bonferroni method are shown.

Projections of movements during walking into the RDS clearly distinguished between movements of healthy versus PD participants. As shown in Fig 2A, projections into the RDS show that the average walking movements from PD participants were clearly separable from movements of healthy participants (at least in one of the projections, U-test, p < 0.05 corrected by 12–6 sensors and 2 conditions). Movement statistics showed that projections of all movements aligned with either left or right steps were able to discriminate healthy with respect to PD participants in at least one of the RDS projections with p < 0.05 (U-test), with the exception of right wrist movements aligned with the right steps. In addition, we fed these movement projections into 4 different classifiers (logistic regression, decision trees, random forest, and Naïve Bayes) to evaluate their ability to discriminate PD from healthy participants. All classifiers were able to achieve high accuracy, with the best results obtained using logistic regression and random forest (see Tables 1 and S1). We were also able to estimate PD severity from an estimate of motor impairment (based on the UPDRS total score) with r^2^ values up to 0.41 using the acceleration signal (see Fig 2C-Acceleration). When velocity was used instead of acceleration to generate the movement profile, the correlation results with the UPDRS scores were slightly better reaching r^2^ values up to 0.46 (see results for right wrist during right-foot steps in Fig 2C-Velocity). Furthermore, we obtained a higher r^2^ of 0.54 (at right wrist during right-foot steps) when we combined profiles obtained from velocity and acceleration.

**Table 1:** Discrimination results of healthy vs PD participants for using acceleration, velocity and the combination of both.

Position	Trigger	Acceleration					Velocity					Acceleration + Velocity				
		Best Classifier	Accuracy	AUC	Precision	F1	Best Classifier	Accuracy	AUC	Precision	F1	Best Classifier	Accuracy	AUC	Precision	F1
Left Wrist	Left	LR	0.83	0.68	0.28	0.02	LR	0.82	0.70	0.29	0.03	LR	0.82	0.70	0.29	0.03
Right Wrist		LR	0.83	0.67	0.27	0.02	LR	0.83	0.66	0.23	0.00	LR	0.83	0.66	0.23	0.00
Left Foot		RF	0.86	0.84	0.63	0.55	LR	0.83	0.72	0.28	0.03	LR	0.83	0.72	0.28	0.03
Right Foot		RF	0.86	0.85	0.63	0.55	LR	0.84	0.65	0.29	0.00	LR	0.84	0.65	0.29	0.00
Lumbar		LR	0.81	0.79	0.34	0.19	LR	0.81	0.77	0.34	0.08	RF	0.82	0.88	0.63	0.54
Sternum		LR	0.83	0.76	0.36	0.17	LR	0.81	0.76	0.30	0.04	LR	0.81	0.76	0.30	0.04
Left Wrist	Right	LR	0.81	0.71	0.35	0.11	LR	0.81	0.70	0.28	0.01	LR	0.81	0.70	0.28	0.01
Right Wrist		LR	0.83	0.63	0.23	0.00	LR	0.83	0.62	0.21	0.00	LR	0.83	0.62	0.21	0.00
Left Foot		LR	0.84	0.73	0.46	0.14	RF	0.83	0.79	0.54	0.46	RF	0.83	0.79	0.54	0.47
Right Foot		RF	0.83	0.86	0.66	0.58	LR	0.82	0.76	0.33	0.04	LR	0.82	0.76	0.33	0.04
Lumbar		RF	0.82	0.88	0.66	0.57	RF	0.83	0.90	0.72	0.64	RF	0.83	0.90	0.73	0.64
Sternum		RF	0.85	0.89	0.71	0.62	RF	0.83	0.89	0.69	0.61	RF	0.83	0.89	0.69	0.61

The best results among 4 classifiers (logistic regression—LR, decision trees—DR, random forest—RF, and Naïve Bayes—NB) were reported in terms of accuracy, AUC, precision and F1-score.

### Personalized single body part kinematics with medication states (ON vs. OFF)

**Fig 3:**
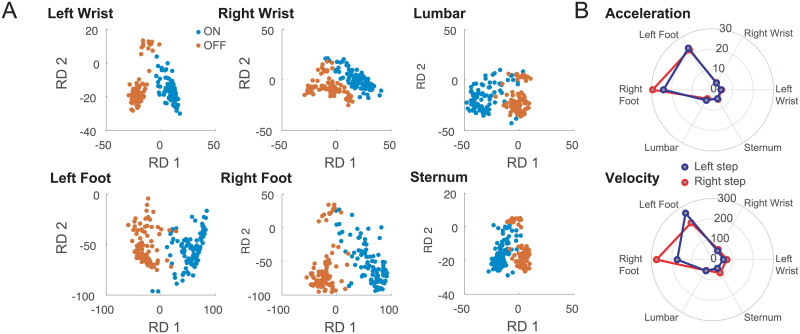
Discrimination of ON versus OFF states. **A**–Example of projecting acceleration data for each body part in both medication states (ON in blue and OFF in orange) using participant-specific templates. **B**–Mean population differences of ON vs. OFF medication states by sensor position triggered either by left (red) or right step (blue). The same analysis was repeated both for acceleration (upper) and velocity (lower) signals.

To understand if the proposed solution would discriminate movements in the same body part in ON versus OFF medication states, we created a personalized model for each participant and each body part (see Methods). Fig 3A shows an example of steps from one participant projected into a 2D RDS for each of the body parts for ON and OFF medication states. It can be seen that movements appear to be completely different at each body part. Table 2 shows the percentage of PD participants in which there is a significant difference between medication states for all the analyzed body parts. Overall, we could significantly distinguish ON vs. OFF in at least 85% of the participants. Percentage values obtained using velocity were slightly better than acceleration, ranging from 88% to 100% (see Table 2 for the complete breakdown). Although the model built for each participant is very sensitive to differences between ON-OFF, those differences are also visible at a population level (Fig 3B). For population comparisons we used a generalized model built on the healthy participants in order to have a common reference (as done for the previous analysis).

**Table 2:** Summary of 33 PD participants during the discrimination of ON versus OFF state by sensor position triggered either by left or right step. The same analysis was repeated both for acceleration (left) and velocity (right).

	Velocity		Acceleration	
	Left Step	Right Step	Left Step	Right Step
Left Wrist	30/33 (90.91%)	30/33 (90.91%)	32/33 (96.97%)	30/33 (90.91%)
Right Wrist	30/33 (90.91%)	28/33 (84.85%)	30/33 (90.91%)	29/33 (87.88%)
Left Foot	33/33 (100%)	32/33 (96.97%)	31/33 (93.94%)	32/33 (96.97%)
Right Foot	32/33 (96.97%)	31/33 (93.94%)	32/33 (96.97%)	33/33 (100%)
Lumbar	31/33 (93.94%)	33/33 (100%)	31/33 (93.94%)	33/33 (100%)
Sternum	31/33 (93.94%)	32/33 (96.97%)	32/33 (96.97%)	31/33 (93.94%)

### Body symmetries and coordination in PD progression

**Fig 4:**
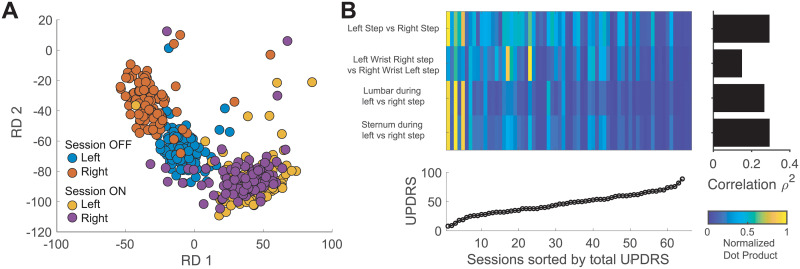
Effects of PD severity on movement symmetry during walking. **A**–Projection of the steps from one participant—accelerometer traces shown in S3 Fig—on the template built on healthy participants during ON versus OFF medication states. **B—**Similarity of the average projections with respect to different body parts and correlation with the score of total impairment.

Finally, we wanted to quantify the relationship between the left-right symmetry of body movements (see for example S3 Fig). Fig 4A shows an example of projecting movement for both feet at each step during ON vs. OFF sessions. It is noticeable that when the participant was ON, both left and right feet had a similar movement pattern (i.e. overlapping clusters), while in the OFF state, movement patterns in both feet are clearly different. Furthermore, left foot movements appeared to have greater differences than right foot. When symmetrical behavior in terms of similarity (see Methods) was plotted against the motor impairment scores (Fig 4B), a significant correlation of the symmetry of the steps and core (both trunk and sternum) during left versus right steps emerged (r^2^ = 0.36, p < 0.001, n = 66). Similarly, lumbar and trunk movements during left and right steps produced a significant estimation of disease severity (lumbar r^2^ = 0.31, sternum r^2^ = 0.34, p < 0.001). A weaker correlation was observed in the symmetrical behavior of the arm swing as captured by wrist movements (r^2^ = 0.13, p = 0.0139, uncorrected).

## Discussion

In this work, we presented a technique for capturing temporal profiles of body movement during walking from wearable sensor data. When the method is applied to people with PD, we showed how coordinated and symmetric behavior is affected by disease severity. The sensitivity of the method is such that: 1) it can discriminate self-reported medication states at each body part, 2) it provides a measurement of motor impairment proportional to disease severity.

Asymmetric deficits are a well-known feature of PD [30]. For example, Sant’Anna et al. [27] achieved high AUC classification of healthy participants from participants with PD by calculating a symmetry index using sensors to determine if symmetry of both upper and lower limbs of 11 early-to-mid-stage PD participants while walking is different from 15 control participants. In agreement with Sant’Anna, our method is able to distinguish people with PD from healthy participants with a very high accuracy and at the same time, to provide a proxy for medication state and disease severity. This result was achieved because we used step movements to characterize body coordination. In fact, when we tested these methods using random events during walking as reference to build the RDS, the movement projections showed neither significant difference between healthy and PD participants (p > 0.05, U-Test) nor correlation for PD participants of the RDS with respect to the UPDRS score (p > 0.05). It should be noted that determining the difference between healthy and people with Parkinson’s is outside the scope of this paper since the healthy subject cohort was neither age matched nor evaluated with the UPDRS to assess a lack of pathological signs of PD. Nevertheless, given those preliminary observations we expect the method to be generalizable to detect early stages of divergence from healthy behavior.

Specifically for disease progression, we found only a few specific metrics for gait assessment that correlated with UPDRS III scores. For example, Rodríguez-Molinero et al. [31] found a correlation r^2^ = 0.31, p < 0.001 with the frequency content of strides during walking. Better correlations were found when the metrics were compared with UPDRS motor subscores for gait and balance such as angular velocity [32] in both early PD (r^2^ = 0.17, p < 0.01) and PD + freeze of gait participants (r^2^ = 0.61, p < 0.01). For EMG data, Spasojević et al. [14] obtaining significant correlations between their estimated features and UPDRS scores (r^2^ > 0.25) by analyzing the movement of the arm/hand in 17 PD participants. In a study more aligned with ours, Huang et al. [11] explored body coordination of the arms in 8 PD participants while walking. In their study, they found high correlation between their estimated features and the UPDRS scores of limbs (equal to the sum of UPDRS akinetic/rigidity and tremor scores of upper and lower extremities) r^2^ = 0.58, p = 0.049 for all limbs, and r^2^ = 0.69, p = 0.021 for most affected limbs. Our results are consistent with these previous publications as we obtained high correlation with UPDRS III total scores. The strength of our study relative to previous work is a larger study population (33 PD), the use of wearable sensors that make at-home monitoring feasible, and the inclusion of body parts (e.g. feet) whose movement is more stereotypical in everyday walking than wrist movement.

Our approach better characterized lower limb symmetry compared to movement descriptions made by aggregating measurements such as step length, and swing velocity as a way to assess disease progression. For example, while Lewek et al. [12] showed that lower limb symmetry was not significantly different across 12 participants using arm swing magnitude, stride time, and side-to-side asymmetry in a video recording, our study shows that the lower limb coordination (analyzed only at each step while walking) is increasingly impaired with disease severity, as indicated by a high correlation (r^2^ = 0.36) of this measure with the total UPDRS score. This suggests that lower limb movement can be used to monitor PD severity when steps can be identified (see also Del Din et al. [19]). In fact, Penko et al. [33], in their study of lower extremity coordination patterns in people with PD during cycling, suggested that the UPDRS sub scores of lower extremities are not sensitive enough to estimate lower limb function.

When we analyzed body coordination by checking symmetry through vector similarity, we found that similarity was higher for participants with lower UPDRS score (see intensity plot in Fig 4B). Therefore, as disease severity increases (higher UPDRS scores), the symmetry between limbs decreases. This behavior is very pronounced for feet, sternum and lumbar areas but not for arm swing (wrist movement, bar plot in Fig 4B) in spite of its vital role in maintaining stability during gait [34]. This finding is supported by the fact that arm swing asymmetry is one of the earliest signs of PD [35], and it is reflected by the low similarity distance obtained for participants with low UPDRS scores (intensity plot in Fig 4B). In other words, wrist similarity deteriorates early (lower UPDRS scores) and remains poor as participants progress to higher scores.

Among our findings, we observed that results obtained with velocity were slightly better than acceleration. Our interpretation was that since walking triggers ballistic movement, the change in velocity is better suited to capture changes in PD during walking [36].

In addition to its good performance, the proposed methodology has two main advantages: simplicity and scalability. Since we only used accelerometer data, our method is well-suited for low-power devices that can be embedded in clothes, and/or wearables. In fact, most consumer-grade wearables collect accelerometer data, which can be used to apply our methodology. In addition, our method is based on unassisted walking, which can be performed in most locations. Therefore, we feel that our proposed methodology could be easily deployed for longitudinal studies in large cohorts of patients in their home settings without complex instructions or scripted tasks. Furthermore, we show that our method is also able to detect divergence from healthy movement, which could be used to assess other neurodegenerative diseases affecting movement or having a movement phenotype.

Although completely speculative because of the lack of physiological correlates, changes in gait during walking in PD might be related to basal ganglia connections to the brainstem, which control different aspects of movement initiation and control of locomotion [37,38]. Indeed, the Peduncolopontine nucleus in the brainstem is the major relay of the basal ganglia to spinal and cerebellar nuclei for the control of locomotion [39] and skill learning [40]. Recent experimental [38,41] and computational [42] studies have shown that manipulations of this pathway have effects on both the initiation and control of walking, suggesting that progressive degeneration in these connections could result in alteration of the commands for movement coordination. Indeed, those regions are targets for deep brain stimulation interventions aimed at restoring motor functions in people with PD [43].

Limitations of this work include the number of participants, which although greater than typical studies in the field, is still not big enough to generalize to all the possible variants of PD phenotypes, and the cross-sectional nature of the experiment. However, given that our method incorporates clinical intuition by analyzing the continuous nature of the movement, and that our results show high correlation with UPDRS scores and can accurately distinguish medication states, we feel that the proposed method could be used as a candidate digital biomarker for prospective or longitudinal studies. In addition, our methodology is not restricted to Parkinson’s disease and can be used for monitoring other neurological diseases that affect body symmetry during walking inside and outside clinical settings.

In summary, we have demonstrated a method based on movement templates using accelerometry from different parts of the body that can be used as a surrogate to evaluate disease severity and identify ON vs. OFF medication states. This method has the potential to generate novel digital biomarkers for PD.

## Methods

### Participants

This study was approved by the Tufts Health Sciences Campus Institutional Review Board (IRB number 12371) and it was conducted at the Tufts Medical Center, Boston, Massachusetts. All participants were over 18 years of age and gave their written, informed consent prior to the start of the study. All of the methods were carried out in accordance with the relevant guidelines and regulations as documented in the IRB submission. All PD participants had a clinical diagnosis of idiopathic Parkinson’s disease consistent with the United Kingdom (UK) Parkinson’s Disease Society Brain Bank Clinical Diagnostic Criteria. All responded to L-DOPA treatment and were able to recognize “wearing-off” symptoms. Exclusion criteria consisted of psychiatric illness that would interfere with the tasks, other neurological diseases, treatment with an investigational drug within 30 days or 5 half- lives (whichever was longer) preceding the enrollment in this study, alcohol consumption exceeding 7 drinks/week for females or 14 drinks/week for males, and participants with cardiac pacemakers, electronic pumps or any other implanted medical devices (including deep brain stimulation devices). More details about the study can be found in [44].

Healthy participants were recruited, and the protocol was run at IBM and Pfizer sites. The study protocol was approved by the Schulman Independent Institutional Review Board (now Advarra) IRB # 201500837. All participants were over 18 years of age and gave their written, informed consent prior to the start of the study. The protocol was carried out in accordance with the relevant guidelines and regulations documented in the IRB submission.

We included in this analysis only data from 31 healthy participants and 33 PD participants having reliable data from all 6 sensors (see Recording Protocol). Due to technical malfunctions, a participant was excluded when one or multiple sensors did not record any data. Participants with PD were diagnosed in stages 1 (N = 2), 2 (N = 24), or 3 (N = 7) of the Hoehn and Yahr scale [45]. Table 3 provides demographic and clinical information of both cohorts. All participants were able to walk unaided. All evaluations (Hoehn and Yahr, UPDRS) were performed by one of the authors who is a neurologist specializing in movement disorders (Dr. Ho).

**Table 3:** Demographics and clinical information of the participants.

	PD participants	Controls
**Number of** participants	33	31
**Age**	69 ± 8 years	49±9 years
**Gender (%male)**	49%	67%
**Height**	171 ± 9 cm	175 ± 10 cm
**Weight**	84 ± 24 Kg	79 ± 18 Kg
**Education level (post-graduate)**	49%	77%
**Dominant hand (%right)**	90%	88%
**Disease duration**	6 ± 4 years	--
**Daily levodopa dose**	380 ± 304 mg	--
**UPDRS part III (ON/OFF)**	40 ± 17 / 54±16	--
**UPDRS Gait (ON/OFF)**	1.03 ± 0.95 / 1.45 ± 0.97	--
**UPDRS Posture Stability (ON/OFF)**	1.36 ± 1.11/ 1.76 + 0.90	--
**Hoehn and Yahr scale**	2.15 ± 0.51	--
**Clinical symmetry (Right/Left/None)**	13/18/3	--
**Asymmetry index: |L-R|/(L+R)**	0.19 ± 0.17	--

### Recording protocol

Each participant underwent two sessions. In one session, data was collected after participants took their usual dopamine replacement therapy and were self-reportedly (and confirmed by the neurologist) in the ON-state. In the other session, data was collected when participants were in the self-reported (and confirmed by the neurologist) OFF-state, meaning their medication had washed out. The order of the sessions was randomized among participants. To ensure that data acquired from PD participants in ON-state were acquired during peak effects, all participants arrived in OFF-state to the clinic. They took their scheduled dopamine dose and began the ON evaluation after confirmation by both participant and neurologist (state ON/OFF questioning was performed every 0.5 hours until ON or 1.5 hours post-dose, whatever was earlier). If the first session was in ON state, the second session began between 0.5 to 1 hour before their next scheduled levodopa dose the same day or up to 14 days later.

In each session the motor examination of the UPDRS was administered by the neurologist. It was comprised of 33 sub-scores based on 18 items, several with right, left or other body distribution scores. In addition, a set of tasks simulating various activities of daily living (ADLs) were performed. As part of the gait tasks, participants performed an instrumented walking task where they walked 10 meters at their normal pace back and forth in a closed hospital corridor as shown in Fig 1A for a duration of 31.32 ± 29.71 seconds for healthy and 120.94 ± 5.27 seconds for PD participants (see [46] for details).

We used Opal Version 1 wearable sensors (APDM Wearable Technologies) to capture body part movement. These devices record acceleration, angular velocity, and magnetic flux density. Mancini et al. [47] have studied the reliability of these sensors by assessing temporal and spatial measurement showing good reliability with an intra class coefficient higher than 0.55. In our study we used acceleration measurements recorded at 128 Hz. To cover most of the body, these sensors were located on 6 different body parts as shown in Fig 1C (see also S1 Fig for location of sensors and orientations): both feet, both wrists, lumbar and sternum. Informed consent from one of the authors was obtained for the use of the picture illustrating sensor placement in S1 Fig.

### Clinical variables

The UPDRS motor examination (part 3) score can be broken down into five main categories: speech, facial expression, bradykinesia, tremor, and postural instability and gait disorder (PIGD). In this work, we use the total UPDRS-part 3 score because it better captures the overall disease severity. Average total UPDRS for all PD participants 40 ± 17 and 54 ± 16 for ON and OFF state, respectively.

### Data analysis and statistics

Natural walking results from a pendulum motion of the body over each leg as the ankle dorsiflexes during the stance phase. Displacement of the center of mass during a step produces an unstable equilibrium and different parts of the body need to interact in a coordinated way to prevent an individual from falling [48]. At each step, synchronization can be observed at both core/shoulder (measured at lumbar and sternum level), upper (measured at the wrists level) and lower extremities (measurable at the feet level). Movements at each step have a complex 3-dimensional (3D), stereotypical behavior that we analyzed at 6 different body positions using sensors with three-axis accelerometer signals. As it was mentioned before, the scripted walking task included steps and turnings (see Fig 1A). However, in this study, we did not include turning since these are asymmetric movements with different dynamics than natural walking [27].

Sensor data was initially filtered to remove unwanted noise using a 2^nd^ order band-pass Butterworth filter with low cut-off frequency = 1Hz and high cut-off frequency = 10 Hz. To identify the interval for each step (right and left), the acceleration-magnitude detector algorithm was used. Here, steps are identified based on a threshold for the acceleration of the foot of more than 2 m/s^2^ in the sagittal plane. This threshold captured the start of the heel lift phase (see Fig 1B). This algorithm is similar to the zero-velocity algorithm which uses a combination of both accelerometer and gyroscope [49]. We considered a step successful if its duration was greater than 625 ms. Furthermore, for each foot, we performed a principal component analysis (PCA) on the sagittal acceleration profile: steps in which the first projection was greater than the projection average minus its standard deviation were excluded. This criterion allowed us to identify most of the steps during rotational movement which did not follow a stereotyped dorsiflexion sequence. We validated the procedure to identify steps by visual inspection from videos recorded during the task. Once each step start was identified, the temporal signal for the accelerometer for the timeframe from 250 ms before to 1000 ms after the event was extracted for further analysis. Velocity was obtained by cumulative trapezoidal numerical integration of the accelerometer profiles for each timeframe.

For our study of walking in people with PD, we divided the analysis into two stages: single body part kinematics, and body symmetry and coordination. At each stage, we examined the association between disease severity based on UPDRS score and the effects of dopaminergic replacement therapy on movement.

In most of the cases, data was found to be non-normally distributed and for consistency non-parametric methods were used through the paper. Non-parametric tests were used (U-test or sign-rank test for paired observations) and Bonferroni corrections for multiple comparisons were adopted when needed. We used linear regression for correlating signals and reported the results in terms of r^2^. A threshold of p = 0.05 for significance was adopted.

#### Single body part kinematics in PD progression

To measure and quantify divergence from stereotypical behavior, we constructed a template of the 3D movement produced by healthy volunteer (HV) participants during left or right steps for each body part. To create those templates, we first created a 1D signal by concatenating the 3 axes (X, Y and Z) of the acceleration signal for each sensor (body part, see S2A and S2B Fig). Then, for each sensor, we generated a matrix in which each row was the time series of steps from the same side (right or left) of each of the 31 healthy volunteers. We applied PCA (see S2A and S2B Fig) to the above matrix to extract the main components of the movements. PCA is an invertible method to decompose the original signal into uncorrelated primitives of movement: PCs (principal components) represent dimensions of the movement of a healthy participant. In general, a limited number of PCs (e.g. 2–3 dimensions) were able to capture most of the variance in continuous movements. In this study, the first 3 PCs accounted between 42 and 70% (average ± std = 58% ± 9%) of the variance of the data (see Fig 2C). Capturing most of the signal allowed us to use most of the intra-subject similarity ignoring the inter-subject variability. Once we obtained the template of the stereotypical movements of the whole population of healthy participants, we projected the movements of PD participants into the same RDS. This allowed us to capture abnormalities with respect to HV. In addition, we use these projections as features to assess disease burden (see Figs 2 and 3). This procedure was performed independently for each step and for each body part, as well as for each type of signal (acceleration and velocity). As shown in S2D Fig, the use of an RDS allowed us to reconstruct movements to inspect the effect of the disease on the movement profile.

As an initial assessment of the capability of the extracted features (projections) to characterize PD phenomenology we performed a U-test (corrected for multiple comparisons) to check if the projections in each of the body parts were different in HV vs. PD participants. To quantify the associations with PD progression, we calculate r^2^ values after performing a linear regression using the projected data in 3 PCs and UPDRS scores.

#### Single body part kinematics relative to medication (ON vs. OFF)

To analyze the effects of dopamine replacement therapy on movements, we modified the previous design to create templates of body part movements for each participant during either ON or OFF medication states. This means that for each participant we generated templates of movements specific to that individual using the above-mentioned approach. By creating participant specific templates, we only need 2 PCs to represent more than 62% of the variance of the data. After projecting the signal on the 2-dimentional template, we compared the distribution of movements of each body part during the ON versus OFF periods.

#### Body symmetry and coordination relative to PD severity

We wanted to quantify the symmetry of body movement to produce a granular metric for subtle changes in body movements. First, we transformed the data to make left and right movements comparable by inverting the axes to project in the same directions. Then we created templates using the same procedure as for single body kinematics (see previous section). To characterize movement symmetry between body parts, we computed a similarity value by calculating the dot product of the average cluster of movements between two body parts. We were interested in four combinations that captured coordination: a) right vs. left foot at right and left steps respectively, b) right wrist at left step vs. left wrist at right step, c) lumbar at right step vs. lumbar at left step, and d) sternum at right step vs. sternum at left step. After computing the similarity for each participant regardless of the session, we computed the r^2^ values between the similarity and the UPDRS scores.

## Supporting information

S1 Fig Coordinate system of the wearable sensor and illustration of their location on the body on one of the authors of the manuscript. (DOCX)

S2 Fig Analysis of sensor data. **A—**Analytic Pipeline. **B—**Three-dimensional data extracted by each sensor at each step (see Methods) were converted to a 1D signal by concatenating the 3 axes (x, y and z). **C**–For each sensor for either left or right steps, we extracted the coefficients of the first three principal components. Projection of the temporal profile on the coefficients of the principle components produced the reduced dimensionality values used in the main analysis. **D—**Reconstruction of the acceleration trajectories (main panel) from movements in the RDS space (inset).
(DOCX)

S3 Fig Acceleration profiles of the left and right foot of a PD participant without (OFF) or after (ON) medication. Values of the acceleration are shown after adjusting signs (see Methods).
(DOCX)

S1 Table Discrimination PD vs. healthy participants by means of logistic regression, decision tree, random forest and Naïve Bayes classifiers with 10-fold cross validation. (DOCX)
