# Supplementary material for: Parkinson’s disease medication state and severity assessment based on coordination during walking
Source: PLoS One. 2021 Feb 17;16(2):e0244842. doi: 10.1371/journal.pone.0244842 (PMC7888646; doi:10.1371/journal.pone.0244842)
Supplement: S1 Fig — (DOCX) [file pone.0244842.s001.docx]

**Supplementary Figure 1 – Coordinate system of the wearable sensor and illustration of their location on the body on one of the authors of the manuscript.**
