# Supplementary material for: Parkinson’s disease medication state and severity assessment based on coordination during walking
Source: PLoS One. 2021 Feb 17;16(2):e0244842. doi: 10.1371/journal.pone.0244842 (PMC7888646; doi:10.1371/journal.pone.0244842)
Supplement: S2 Fig — A—Analytic Pipeline. B—Three-dimensional data extracted by each sensor at each step (see Methods) were converted to a 1D signal by concatenating the 3 axes (x, y and z). C–For each sensor for either left or right steps, we extracted the coefficients of the first three principal components. Projection of the temporal profile on the coefficients of the principle components produced the reduced dimensionality values used in the main analysis. D—Reconstruction of the acceleration trajectories (main panel) from movements in the RDS space (inset). (DOCX) [file pone.0244842.s002.docx]

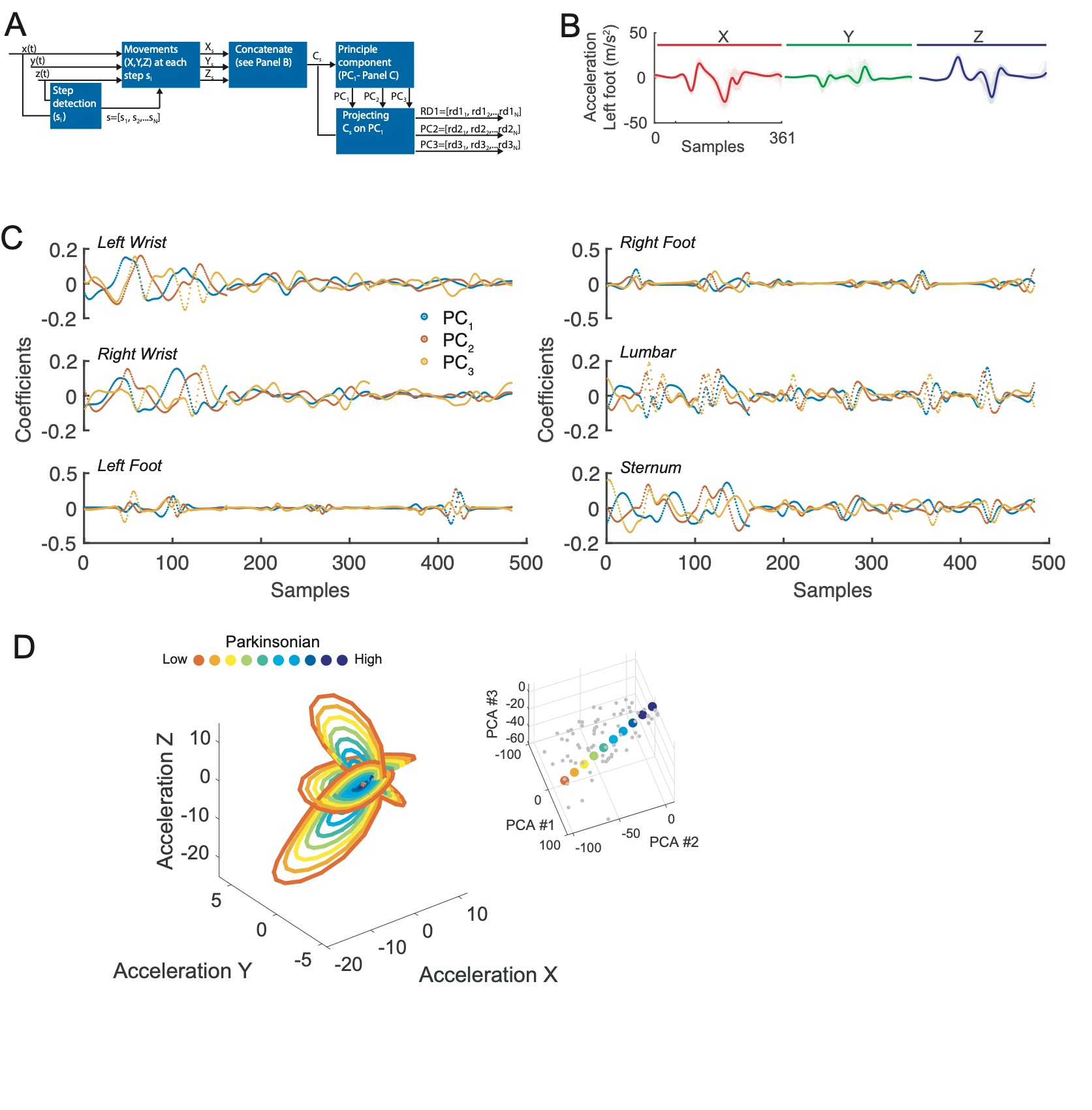


**Supplementary Figure 2 – Analysis of sensor data. A –** Analytic Pipeline. **B –** Three-dimensional data extracted by each sensor at each step (see methods) were converted to a 1D signal by concatenating the 3 axes (x, y and z). **C** **–** For each sensor for either left or right steps, we extracted the coefficients of the first three principal components. Projection of the temporal profile on the coefficients of the principle components produced the reduced dimensionality values used in the main analysis. **D –** Reconstruction of the acceleration trajectories (main panel) from movements in the RDS space (inset).
