# Supplementary material for: Parkinson’s disease medication state and severity assessment based on coordination during walking
Source: PLoS One. 2021 Feb 17;16(2):e0244842. doi: 10.1371/journal.pone.0244842 (PMC7888646; doi:10.1371/journal.pone.0244842)
Supplement: S3 Fig — Values of the acceleration are shown after adjusting signs (see Methods). (DOCX) [file pone.0244842.s003.docx]

**Supplementary Figure 3 – Acceleration profiles of the left and right foot of a PD participant without (OFF) or after (ON) medication. Values of the acceleration are shown after adjusting signs (see Methods).**
