# Supplementary material for: Parkinson’s disease medication state and severity assessment based on coordination during walking
Source: PLoS One. 2021 Feb 17;16(2):e0244842. doi: 10.1371/journal.pone.0244842 (PMC7888646; doi:10.1371/journal.pone.0244842)
Supplement: S1 Table — (DOCX) [file pone.0244842.s004.docx]

| **Classifier** | **Position** | **Trigger** | **Acceleration** | | | | **Velocity** | | | | **Acceleration + Velocity** | | | |
| --- | --- | --- | --- | --- | --- | --- | --- | --- | --- | --- | --- | --- | --- | --- |
|  |  |  | **Accuracy** | **AUC** | **Precision** | **F1** | **Accuracy** | **AUC** | **Precision** | **F1** | **Accuracy** | **AUC** | **Precision** | **F1** |
| Logistic Regression | Left Wrist | Left | 0.83 | 0.68 | 0.28 | 0.02 | 0.82 | 0.70 | 0.29 | 0.03 | 0.82 | 0.70 | 0.29 | 0.03 |
|  | Right Wrist |  | 0.83 | 0.67 | 0.27 | 0.02 | 0.83 | 0.66 | 0.23 | 0.00 | 0.83 | 0.66 | 0.23 | 0.00 |
|  | Left Foot |  | 0.84 | 0.66 | 0.30 | 0.00 | 0.83 | 0.72 | 0.28 | 0.03 | 0.83 | 0.72 | 0.28 | 0.03 |
|  | Right Foot |  | 0.84 | 0.67 | 0.36 | 0.04 | 0.84 | 0.65 | 0.29 | 0.00 | 0.84 | 0.65 | 0.29 | 0.00 |
|  | Lumbar |  | 0.81 | 0.79 | 0.34 | 0.19 | 0.81 | 0.77 | 0.34 | 0.08 | 0.81 | 0.77 | 0.34 | 0.08 |
|  | Sternum |  | 0.83 | 0.76 | 0.36 | 0.17 | 0.81 | 0.76 | 0.30 | 0.04 | 0.81 | 0.76 | 0.30 | 0.04 |
|  | Left Wrist | Right | 0.81 | 0.71 | 0.35 | 0.11 | 0.81 | 0.70 | 0.28 | 0.01 | 0.81 | 0.70 | 0.28 | 0.01 |
|  | Right Wrist |  | 0.83 | 0.63 | 0.23 | 0.00 | 0.83 | 0.62 | 0.21 | 0.00 | 0.83 | 0.62 | 0.21 | 0.00 |
|  | Left Foot |  | 0.84 | 0.73 | 0.46 | 0.14 | 0.83 | 0.66 | 0.40 | 0.00 | 0.83 | 0.66 | 0.40 | 0.00 |
|  | Right Foot |  | 0.81 | 0.80 | 0.43 | 0.22 | 0.82 | 0.76 | 0.33 | 0.04 | 0.82 | 0.76 | 0.33 | 0.04 |
|  | Lumbar |  | 0.82 | 0.78 | 0.41 | 0.22 | 0.80 | 0.79 | 0.40 | 0.15 | 0.80 | 0.79 | 0.40 | 0.15 |
|  | Sternum |  | 0.85 | 0.78 | 0.46 | 0.32 | 0.82 | 0.77 | 0.37 | 0.16 | 0.82 | 0.77 | 0.37 | 0.17 |
| Decision Tree | Left Wrist | Left | 0.75 | 0.65 | 0.25 | 0.40 | 0.74 | 0.63 | 0.23 | 0.37 | 0.73 | 0.63 | 0.23 | 0.37 |
|  | Right Wrist |  | 0.75 | 0.63 | 0.24 | 0.38 | 0.73 | 0.64 | 0.24 | 0.38 | 0.73 | 0.63 | 0.23 | 0.37 |
|  | Left Foot |  | 0.79 | 0.70 | 0.31 | 0.48 | 0.75 | 0.69 | 0.30 | 0.46 | 0.75 | 0.69 | 0.30 | 0.47 |
|  | Right Foot |  | 0.79 | 0.70 | 0.31 | 0.49 | 0.77 | 0.63 | 0.24 | 0.38 | 0.76 | 0.64 | 0.24 | 0.38 |
|  | Lumbar |  | 0.78 | 0.69 | 0.30 | 0.47 | 0.78 | 0.69 | 0.31 | 0.48 | 0.78 | 0.70 | 0.31 | 0.48 |
|  | Sternum |  | 0.78 | 0.68 | 0.29 | 0.45 | 0.77 | 0.68 | 0.28 | 0.45 | 0.77 | 0.68 | 0.28 | 0.45 |
|  | Left Wrist | Right | 0.74 | 0.64 | 0.25 | 0.39 | 0.70 | 0.62 | 0.24 | 0.36 | 0.70 | 0.62 | 0.24 | 0.36 |
|  | Right Wrist |  | 0.72 | 0.63 | 0.25 | 0.38 | 0.73 | 0.63 | 0.25 | 0.39 | 0.73 | 0.63 | 0.25 | 0.39 |
|  | Left Foot |  | 0.78 | 0.68 | 0.31 | 0.47 | 0.76 | 0.65 | 0.27 | 0.42 | 0.75 | 0.65 | 0.27 | 0.42 |
|  | Right Foot |  | 0.78 | 0.72 | 0.36 | 0.53 | 0.75 | 0.67 | 0.29 | 0.45 | 0.75 | 0.67 | 0.29 | 0.45 |
|  | Lumbar |  | 0.79 | 0.71 | 0.34 | 0.51 | 0.78 | 0.74 | 0.39 | 0.56 | 0.78 | 0.74 | 0.39 | 0.56 |
|  | Sternum |  | 0.79 | 0.73 | 0.37 | 0.55 | 0.78 | 0.72 | 0.36 | 0.53 | 0.78 | 0.72 | 0.36 | 0.53 |
| Random Forest | Left Wrist | Left | 0.82 | 0.80 | 0.53 | 0.46 | 0.80 | 0.81 | 0.48 | 0.40 | 0.80 | 0.81 | 0.48 | 0.40 |
|  | Right Wrist |  | 0.81 | 0.80 | 0.47 | 0.40 | 0.79 | 0.81 | 0.48 | 0.41 | 0.80 | 0.81 | 0.48 | 0.40 |
|  | Left Foot |  | 0.86 | 0.84 | 0.63 | 0.55 | 0.81 | 0.85 | 0.61 | 0.52 | 0.82 | 0.85 | 0.61 | 0.52 |
|  | Right Foot |  | 0.86 | 0.85 | 0.63 | 0.55 | 0.81 | 0.78 | 0.48 | 0.40 | 0.81 | 0.78 | 0.48 | 0.39 |
|  | Lumbar |  | 0.81 | 0.87 | 0.57 | 0.51 | 0.82 | 0.88 | 0.62 | 0.55 | 0.82 | 0.88 | 0.63 | 0.54 |
|  | Sternum |  | 0.81 | 0.86 | 0.59 | 0.49 | 0.80 | 0.87 | 0.59 | 0.50 | 0.80 | 0.86 | 0.59 | 0.49 |
|  | Left Wrist | Right | 0.80 | 0.81 | 0.54 | 0.43 | 0.77 | 0.79 | 0.46 | 0.36 | 0.78 | 0.79 | 0.46 | 0.36 |
|  | Right Wrist |  | 0.79 | 0.79 | 0.49 | 0.37 | 0.80 | 0.79 | 0.51 | 0.40 | 0.80 | 0.79 | 0.51 | 0.40 |
|  | Left Foot |  | 0.83 | 0.85 | 0.63 | 0.52 | 0.83 | 0.79 | 0.54 | 0.46 | 0.83 | 0.79 | 0.54 | 0.47 |
|  | Right Foot |  | 0.83 | 0.86 | 0.66 | 0.58 | 0.78 | 0.86 | 0.60 | 0.48 | 0.78 | 0.86 | 0.60 | 0.48 |
|  | Lumbar |  | 0.82 | 0.88 | 0.66 | 0.57 | 0.83 | 0.90 | 0.72 | 0.64 | 0.83 | 0.90 | 0.73 | 0.64 |
|  | Sternum |  | 0.85 | 0.89 | 0.71 | 0.62 | 0.83 | 0.89 | 0.69 | 0.61 | 0.83 | 0.89 | 0.69 | 0.61 |
| Naïve Bayes | Left Wrist | Left | 0.82 | 0.77 | 0.35 | 0.25 | 0.79 | 0.73 | 0.27 | 0.05 | 0.79 | 0.73 | 0.27 | 0.05 |
|  | Right Wrist |  | 0.81 | 0.72 | 0.36 | 0.33 | 0.79 | 0.70 | 0.29 | 0.17 | 0.79 | 0.70 | 0.29 | 0.17 |
|  | Left Foot |  | 0.82 | 0.73 | 0.35 | 0.23 | 0.82 | 0.72 | 0.30 | 0.15 | 0.82 | 0.72 | 0.30 | 0.15 |
|  | Right Foot |  | 0.86 | 0.74 | 0.45 | 0.27 | 0.83 | 0.66 | 0.30 | 0.11 | 0.83 | 0.66 | 0.30 | 0.11 |
|  | Lumbar |  | 0.81 | 0.81 | 0.37 | 0.43 | 0.81 | 0.80 | 0.34 | 0.27 | 0.81 | 0.80 | 0.34 | 0.27 |
|  | Sternum |  | 0.81 | 0.78 | 0.34 | 0.33 | 0.77 | 0.79 | 0.31 | 0.19 | 0.77 | 0.79 | 0.31 | 0.19 |
|  | Left Wrist | Right | 0.81 | 0.74 | 0.36 | 0.28 | 0.79 | 0.71 | 0.27 | 0.07 | 0.79 | 0.71 | 0.27 | 0.07 |
|  | Right Wrist |  | 0.81 | 0.69 | 0.33 | 0.27 | 0.79 | 0.67 | 0.28 | 0.11 | 0.79 | 0.67 | 0.28 | 0.11 |
|  | Left Foot |  | 0.83 | 0.76 | 0.47 | 0.32 | 0.83 | 0.71 | 0.36 | 0.14 | 0.83 | 0.71 | 0.36 | 0.14 |
|  | Right Foot |  | 0.83 | 0.81 | 0.50 | 0.37 | 0.81 | 0.77 | 0.35 | 0.18 | 0.81 | 0.77 | 0.35 | 0.18 |
|  | Lumbar |  | 0.81 | 0.81 | 0.39 | 0.40 | 0.79 | 0.80 | 0.36 | 0.22 | 0.79 | 0.80 | 0.36 | 0.22 |
|  | Sternum |  | 0.81 | 0.78 | 0.37 | 0.31 | 0.75 | 0.79 | 0.32 | 0.12 | 0.75 | 0.79 | 0.32 | 0.12 |

**Supplementary Table 1 – Discrimination PD vs. healthy participants by means of logistic regression, decision tree, random forest and Naïve Bayes classifiers with 10-fold cross validation.**
